# A Child with Local Lipohypertrophy following Recombinant Human Growth Hormone Administration

**DOI:** 10.1155/2016/9648043

**Published:** 2016-10-10

**Authors:** Ilan J. N. Koppen, Roel Bakx, Chris C. de Kruiff, A. S. Paul van Trotsenburg

**Affiliations:** ^1^Department of Pediatric Gastroenterology, Emma Children's Hospital, Academic Medical Center, Amsterdam, Netherlands; ^2^Pediatric Surgical Centre Amsterdam, VUMC/Emma Children's Hospital, Academic Medical Center, Amsterdam, Netherlands; ^3^Department of Pediatric Endocrinology, Emma Children's Hospital, Academic Medical Center, Amsterdam, Netherlands

## Abstract

Local lipohypertrophy due to recombinant human growth hormone (rhGH) administration is a rare phenomenon. Here, we report a case of an 11-year-old girl who presented with a paraumbilical swelling, approximately one year after the start of rhGH treatment for short stature due to the presumed diagnosis of partial growth hormone insensitivity. Ultrasound imaging revealed an asymmetric distribution of subcutaneous fat tissue at the rhGH administration site, indicating local lipohypertrophy. After sparing her routine injection site and alternating other sites, the swelling disappeared within 6 months. Although the precise cause of local lipohypertrophy resulting from rhGH administration is still unclear, it might be related to the presumed diagnosis of partial growth hormone insensitivity.

## 1. Introduction

Human growth hormone (hGH) is usually administered via subcutaneous injections. Besides well-known adverse events such as peripheral edema, benign intracranial hypertension, and slipped capital femoral epiphysis, a less known and rare side effect is local lipohypertrophy, a phenomenon with an incompletely understood pathophysiology. Although innocent and easy to treat, it may give rise to concern in patients and caregivers.

## 2. Case

An 11-year-old girl was referred to the department of pediatric surgery for a newly developed swelling on the left side of the umbilicus. The swelling varied in size and was painful at times. Yet there was no history of trauma and there were no signs of inflammation or infection. Physical examination revealed a soft, painless swelling on the left side of the umbilicus (2 × 3 cm). There was no sign of a fascia defect and the Valsalva-manoeuvre was negative.

**Figure 1:**
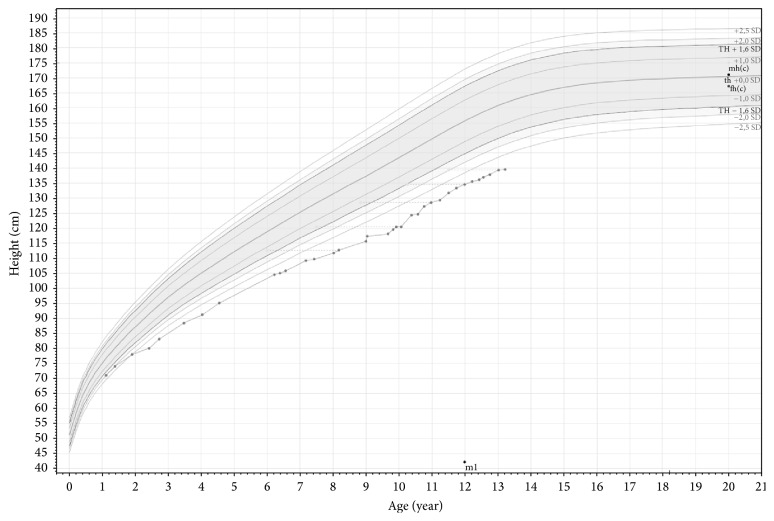
Growth curve (height for age).

The girl had an extensive medical history. Early in life, she suffered from unexplained postnatal hypotonia and respiratory failure, for which extensive investigations were performed. Genetic testing showed a normal female chromosome pattern (46, XX), and DNA testing for 22q11 microdeletion syndrome, Prader-Willi syndrome, and Steinert syndrome was negative. A cause for these transient postnatal problems could not be identified. In addition, she had a right-sided inguinal hernia at birth and a left-sided inguinal hernia at the age of 10 months; both were surgically corrected. At the age of three years, she underwent surgical correction for an umbilical hernia. Because of short stature due to (presumed) partial growth hormone resistance, she was using rhGH since the age of approximately 10 years. When the girl was 2.5 years old, her growth curve had declined (Figure 1). Her height standard deviation (SD) score had decreased from −2.0 at the age of one year to −3.0 at the age of 2.5 years. Her target height SD score was +0.8 SD (height of the mother: 175 cm, height of the father: 180 cm). After ruling out several disorders as possible cause of the deflecting growth (i.e., celiac disease, renal/liver dysfunction, hematological diseases, and systemic/inflammatory diseases), around the age of 3 years GH stimulation testing (arginine and L-dope and propranolol) showed a maximum GH concentration of 59.0 mE/L, ruling out classical growth hormone deficiency. Thyroid function was normal. SHOX and FGFR3 gene mutation analysis, conducted because of mild disproportion (sitting height/height ratio SD score: just > +2.5 SD), and karyotyping to rule out mosaic Turner syndrome revealed no abnormalities. Between the ages of three and seven years, height stabilized around −3 SD. However, between ages seven and ten, growth further deflected to −3.8 SD. Because repeated serum IGF-1 measurement revealed concentrations below −2 SD for age, an IGF-1 generation test was performed (Table 1). After these investigations, she was treated with rhGH (daily subcutaneous injections) under the suspicion of partial GH resistance. During the first year of treatment, growth velocity clearly increased, resulting in a height SD score of −3.3 at the age of 11 years (Figure 1). At the time of presentation, the girl was administering rhGH by a needle-free system, both to the left and to right sides of the umbilicus.

With the aforementioned clinical finding and medical history in mind, the differential diagnosis of the paraumbilical swelling included hernia, local inflammatory/allergic reaction to the medication, lipoma, hemangioma, granuloma, cyst, abscess, and neoplasm. Ultrasound imaging of the swelling was performed, which revealed an asymmetric distribution of subcutaneous fat on the injection site, with a thickness difference of 6 mm (left more than right). There was no sign of hernia. The asymmetric fat distribution at the transjection site led to the suspicion of the rare occurrence of subcutaneous lipohypertrophy. The girl was instructed not to administer rhGH at the site of the swelling anymore and to alternate delivery sites to prevent repeated administration of rhGH at the same site. After 6 months, the swelling had decreased and the pain had disappeared and she had reported no (other) adverse effects.

## 3. Discussion

The availability of rhGH has broadened its range of clinical applications [1]. Nowadays, approved indications for rhGH treatment include growth hormone deficiency (in children and adults), short stature due to Turner syndrome, incomplete catch-up growth in children who were small for gestational age, growth failure associated with chronic renal failure, Prader-Willi syndrome and, more recently, idiopathic short stature (in children), and AIDS-related wasting and fat accumulation associated with lipodystrophy (in adults) [1].

Because rhGH is a peptide hormone, it is administered via subcutaneous injections. Studies on the effects of rhGH treatment on lipid metabolism have shown a general lipolytic effect [2], and local lipoatrophy at injections sites has been described [3, 4]. The opposite effect, occurrence of lipohypertrophy associated with hGH treatment in children, has been described in only two case reports during the past 15 years [5, 6].

The pathophysiology of local lipohypertrophy due to rhGH administration is incompletely understood. GH stimulates the production of IGF-1 in adipocyte precursor cells. The effect of IGF-1, however, depends on its local concentration at the level of the adipocyte in the adipose tissue. Low IGF-1 concentrations have a lipolytic effect, whereas high concentrations, when sufficient to activate insulin receptors, can induce intracellular glucose transport and lipogenesis [5]. Similarly, lipohypertrophy is frequently seen at the injection site in diabetic patients administering insulin via subcutaneous injections [7–9]. On the other hand, local subcutaneous lipohypertrophy is also seen in patients treated with pegvisomant injections, a growth hormone receptor antagonist [10–13]. In these circumstances, lipohypertrophy may be the result of decreased lipolysis due to decreased effects of GH at the level of the adipocyte, while IGF-1 (or insulin) induced lipogenesis persists, potentially causing a disequilibrium between lipolysis and lipogenesis.

In our case, the presumed diagnosis of partial GH insensitivity might be a clue to the cause of the girl's local lipohypertrophy. The swelling may have resulted from a decreased effect of GH on lipolysis (because of the partial GH insensitivity) and increased lipogenesis due to higher local IGF-1 concentrations than before initiation of rhGH administration. Unfortunately, proof (e.g., a genetic defect) of partial GH resistance was not found. In retrospect, the diagnosis of partial GH insensitivity may even be questioned. The rather firm rise of IGF-1 after seven days of treatment with rhGH in a dose of only 0.7 mg per m^2^ per day (Table 1) suggests the possibility of other diagnoses like secretion of GH with a lower than normal bioactivity or GH neurosecretory dysfunction. However, also in these cases it is tempting to speculate that the cause of the local lipohypertrophy is some kind of disequilibrium between lipolysis and lipogenesis.

## 4. Conclusion

In this 11-year-old girl, rhGH treatment for short stature due to a presumed diagnosis of partial GH resistance caused local, paraumbilical subcutaneous lipohypertrophy. By sparing the swelling and alternating rhGH transjection sites, the lipohypertrophy clearly diminished. Caregivers who treat patients with rhGH should be aware of this rare side effect. Although the precise cause of local lipohypertrophy resulting from rhGH administration is still unclear, it might be related to insensitivity local disequilibrium between rhGH induced lipolysis and IGF-1 concentration dependent lipolysis or lipogenesis.

## Figures and Tables

**Table 1:** IGF-1 generation test result and preceding IGF-1 and IGFBP3 concentrations.

Age (years)	IGF-1 (nmol/L)	SD score	IGFBP3 (mg/L)
8.2	8	−2.0	1.6

9.3	9	−2.1	1.9

9.8			
At baseline	12	−1.8	1.8
After 7 days of daily rhGH, at a dose of 0.7 mg per m^2^ BSA	29	0.0	2.5

BSA: body surface area; IGF-1: insulin-like growth factor 1; IGFBP3: insulin-like growth factor binding protein 3; rhGH: recombinant human growth hormone.

## Abbreviations

FGFR3: Fibroblast growth factor receptor 3
GH: Growth hormone
IGF-1: Insulin-like growth factor 1
rhGH: Recombinant human growth hormone
SHOX: Short stature homeobox.
